# Concomitant Lemierre's Syndrome and Base‐of‐Skull Osteomyelitis Caused by *Arcanobacterium haemolyticum* , Complicated by Ceftriaxone‐Associated Cholecystitis: A Case Report

**DOI:** 10.1002/ccr3.73553

**Published:** 2026-09-16

**Authors:** Angus Lane, Alexandra Stewart

**Affiliations:** ^1^ Department of Infectious Diseases Royal Brisbane and Women's Hospital Brisbane Australia; ^2^ School of Clinical Medicine The University of Queensland Brisbane Australia

**Keywords:** *Arcanobacterium haemolyticum*, case report, ceftriaxone, cholecystitis, Lemierre's syndrome, osteomyelitis

## Abstract

Lemierre's syndrome and base‐of‐skull osteomyelitis can occur together in immunocompetent adolescents with *Arcanobacterium haemolyticum* bacteraemia. Prolonged high‐dose ceftriaxone may precipitate symptomatic cholecystitis. Clinicians should consider *A. haemolyticum* as a causative pathogen in invasive head and neck infection, including Lemierre's syndrome and osteomyelitis, even in immunocompetent hosts.

## Learning Points

- Whilst typically associated with pharyngitis in young patients, *Arcanobacterium haemolyticum* can be complicated by severe invasive infections including Lemierre's syndrome and osteomyelitis in immunocompetent young hosts.
- Ceftriaxone use can be associated with the development of symptomatic cholecystitis, cholelithiasis in adult patients, occasionally necessitating cholecystectomy.
- To date, this is the first reported case of concomitant Lemierre's syndrome and osteomyelitis secondary to *Arcanobacterium haemolyticum* .

## 1 Introduction

*Arcanobacterium haemolyticum* is a gram‐positive bacillus, catalase‐negative, non‐motile, non‐spore‐forming facultative anaerobe, with variable β‐haematolytic status [1, 2]. Previously known as *Corynebacterium haemolyticum*, it is a well‐documented cause of pharyngotonsillitis in adolescents [3]. Mackenzie et al. report an *Arcanobacterium haemolyticum* incidence of 2.5% in a group of 15–18 year‐old patients with pharyngitis [4]. Notably, half the patients in this two‐year study with positive throat swabs for *Arcanobacterium* were found to have a rash [4]. Rarely, is it associated with invasive infection; however, sepsis has been reported on a number of occasions in the literature with rare complications of cavitary pneumonia, endocarditis, osteomyelitis, and Lemierre's syndrome [5]. Invasive disease is thought to arise via contiguous spread from the pharyngeal mucosa or haematogenous dissemination, seeding distant sites including the jugular vein, meninges, and bone [1, 3]. Monotherapy with penicillins has been reported as having high rates of treatment success [6]. Diagnosis can be challenging in routine laboratory settings due to the organism's fastidious growth requirements and phenotypic similarity to other coryneforms [3]. *A. haemolyticum* grows optimally on human or horse blood agar in 5%–10% CO_2_ at 37°C, and β‐haemolysis typically requires 48–72 h to develop [3]. As most routine cultures use sheep blood agar and are read at 24 h, the organism is readily overlooked or dismissed as a coryneform contaminant; the advent of matrix‐assisted laser desorption/ionization time‐of‐flight mass spectrometry (MALDI‐TOF MS) has substantially improved species‐level identification in contemporary laboratories [3, 7].

Ceftriaxone, a third‐generation cephalosporin, is associated with cholecystitis as a rare, but recognized complication of therapy [8]. *The Lancet* described it as ‘pseudolithiasis’ as any ultrasonographic cholelithiasis usually resolves upon cessation of ceftriaxone use [9]. Ceftriaxone precipitates as a salt in the gallbladder forming biliary sludge [10, 11]. A recent study reported high levels of pseudolithiasis in elderly patients exposed to ceftriaxone therapy, however, this was asymptomatic in almost all cases [12]. Dose and duration of treatment are considered among the strongest risk factors for pseudolithiasis [12].

## 2 Case History and Examination

A previously healthy 19‐year‐old male presented with a three‐day history of worsening headache unresponsive to non‐steroidal analgesia. He re‐presented within 24 h with fevers, photophobia, neck stiffness, nausea, and vomiting. He denied sore throat, cough, or rash. Three weeks earlier, he had struck his face on a sandbar while diving in brackish water but sustained no significant injury; this was thought to be incidental and not contributory.

On examination, there was no focal neurology. Extraocular movements were noted to be normal. Lateral neck movement was limited by pain. Cardiovascular examination was normal, and chest auscultation revealed a mild inspiratory wheeze. No rash or lymphadenopathy was present.

Fevers persisted for one week, accompanied by debilitating headaches. Ear, Nose and Throat (ENT) review recommended nasal saline washes for concomitant sinusitis. On Day 10 of admission, he developed left hypoglossal and glossopharyngeal nerve palsies. Neurosurgical and ENT review were sought. During the ENT review, it was noted there were left hypoglossal nerve fasciculations consistent with a lower motor neuron deficit. Functional endoscopic sinus surgery (FESS) on Day 10 demonstrated florid left sphenoid sinusitis with pus identified intraoperatively; a wide left sphenoidotomy was performed and pus was sent for microscopy, culture, and sensitivity (MCS) and fungal culture. Right transnasal sphenoidotomy identified no purulent material. After this procedure, headaches and fevers improved. He was subsequently managed through the Hospital‐in‐the‐Home program.

On Day 19 of antibiotic therapy, he developed acute sharp abdominal pain without fever, diarrhea, or urinary symptoms. Ultrasound demonstrated cholecystitis and choledocholithiasis. He underwent laparoscopic cholecystectomy and intra‐operative cholangiogram, which showed a dilated common bile duct. Histology confirmed chronic cholecystitis with calcified debris.

## 3 Differential Diagnosis, Investigations and Treatment

Initial blood tests showed neutrophilia (22.64 × 10^9^/L, reference range 4.0–11.0 × 10^9^/L) and raised inflammatory markers (C‐reactive protein (CRP) 109 mg/L, reference range < 5.0 mg/L). Liver function tests were normal. Lumbar puncture revealed White blood cell (WBC) 1 × 10^6^/L (reference range < 5.0 × 10^6^/L) and Red blood cell (RBC) 27 × 10^6^/L (reference range < 5.0 × 10^6^/L) with no organisms on Gram stain. No throat culture was obtained. Four blood culture bottles grew *A. haemolyticum* . Two blood culture sets were collected simultaneously at 13:00 h on day one of admission. All four bottles, both aerobic and anaerobic from each set, grew *A. haemolyticum* , with time‐to‐positivity of 20.0 and 19.6 h for the aerobic bottles and 22.9 and 25.4 h for the anaerobic bottles respectively. Repeat blood cultures on day three were negative. The organism was identified by MALDI‐TOF (VITEK MS, bioMérieux) with a score of 99.9%. Samples were inoculated on Muller Hinton (MH) fastidious agar with 5% defibrinated horse blood and inoculated with 0.5 McFarland standard colony suspension. These were incubated in air at 35 degrees Celsius for 24 h. Interpretation was via Clinical and Laboratory Standards Institute (CLSI) breakpoints (M45‐ED3:2016‐ coryneform organisms) [13]. Antibiotic susceptibility testing (AST) was performed by gradient diffusion strips (ETEST–bioMérieux). AST showed the organism was susceptible to penicillin G (Minimum inhibitory concentration (MIC) 0.008 mg/L), ceftriaxone (MIC 0.064 mg/L), clindamycin (MIC 0.032 mg/L), vancomycin (MIC 0.5 mg/L), and ciprofloxacin (MIC 0.5 mg/L). Intraoperative swabs from the left sphenoid sinus and left middle meatus yielded normal skin flora, with no organisms on Gram stain and 2+ leucocytes; Acid Fast Bacillus (AFB) was smear and culture negative.

**FIGURE 1:**
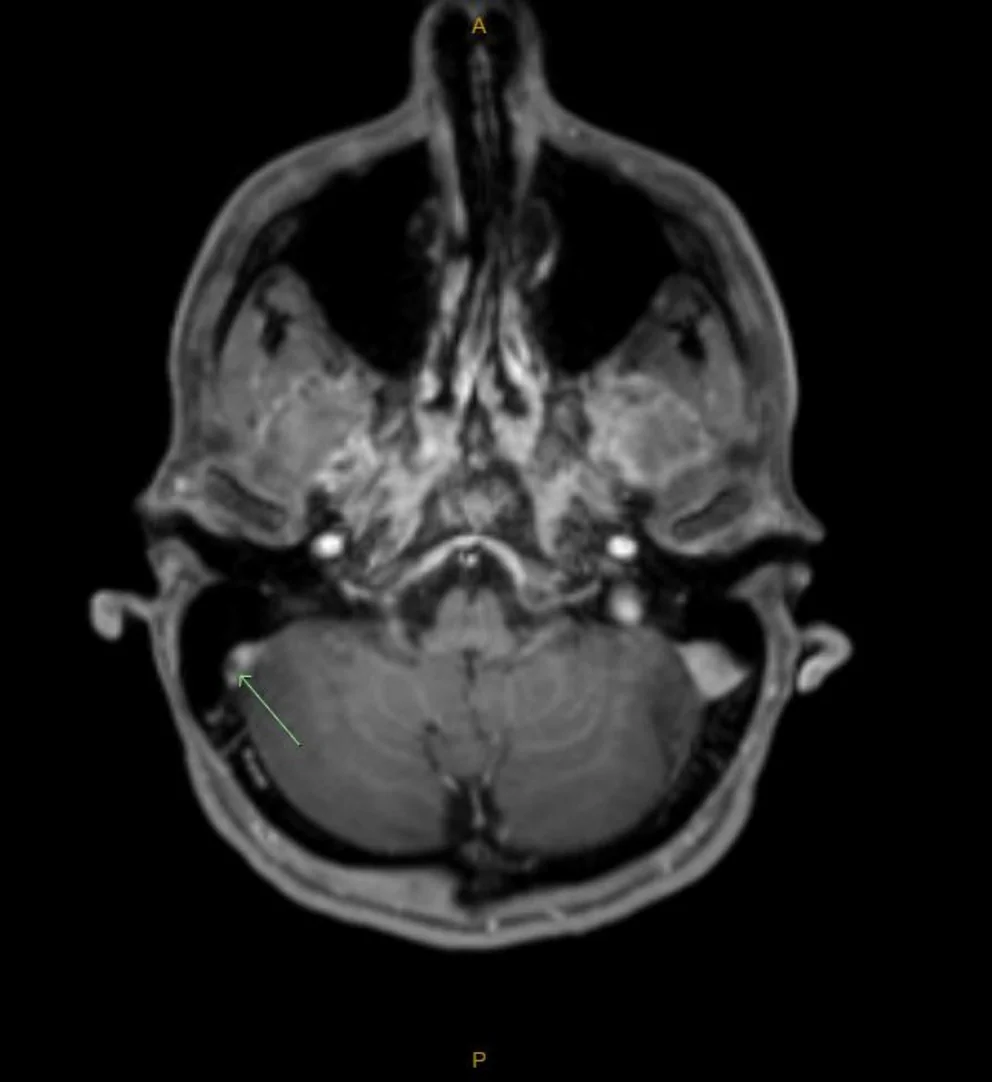
Coronal Section demonstrating a small non‐occlusive thrombus is present within the proximal left internal jugular vein, with a suspected tiny non‐occlusive thrombus within the right sigmoid sinus. No spondylodiscitis or epidural collection identified.

**FIGURE 2:**
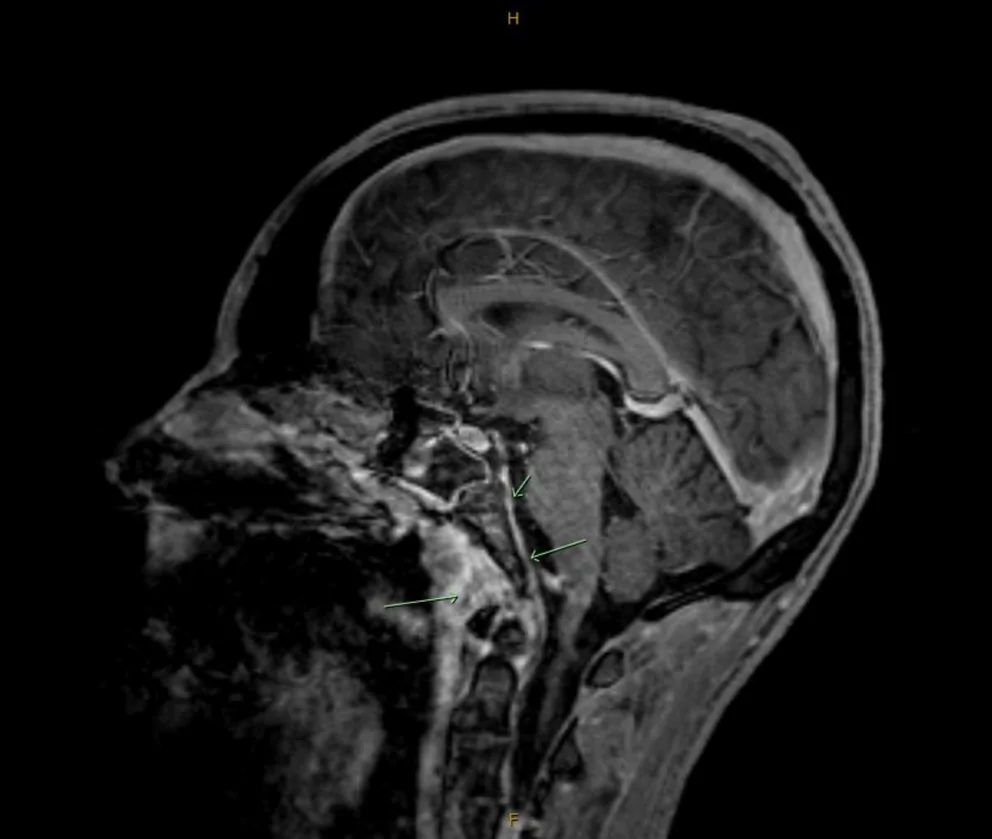
Sagittal Section shows extensive inflammatory change centred around the adenoid and palatine tonsils with associated enhancing soft tissue oedema within the retropharyngeal space and a discrete sliver of rim‐enhancing fluid consistent with a small abscess. Intracranial extension with increased dural enhancement along the clivus consistent with pachymeningitis; no cerebritis or cerebral abscess formation is seen.

Magnetic resonance imaging (MRI) brain and spine (Day 3, Figures 1 and 2) demonstrated inflammatory changes around the adenoid and palatine tonsils, retropharyngeal oedema with a small abscess, intracranial extension with dural enhancement consistent with pachymeningitis, and clival enhancement concerning for early osteomyelitis. A small non‐occlusive thrombus was present in the left internal jugular vein, with possible right sigmoid sinus thrombus. Computed tomography (CT) chest showed no pulmonary septic emboli. CT venogram (Day 6) showed no progression of thrombosis but marked sinus opacification.

A trough ceftriaxone level on Day 5 of therapy was 5.7 mg/L (target 1–20 mg/L). At onset of abdominal pain, blood tests showed normal lipase, bilirubin, transaminases, and inflammatory markers. Ultrasound demonstrated gallbladder wall thickening, pericholecystic oedema, multiple small calculi, and two 6‐mm calculi in the distal common bile duct.

Ceftriaxone and aciclovir were commenced on presentation to cover meningoencephalitis. As the LP excluded bacterial meningitis, the ceftriaxone was continued at 2 g daily and increased to 2 g twice daily when osteomyelitis was suspected. Choice of antibiotic was influenced by need for empiric coverage of culpable organisms, compatibility with Hospital‐in‐the‐Home, and need for penetration into the cerebrospinal fluid (CSF). Empiric metronidazole was not added to the antibiotic regimen as Lemierre's syndrome was diagnosed on Day 3 of admission following identification of left internal jugular vein thrombus on imaging, and at this timepoint *Arcanobacterium haemolyticum* had been isolated from blood cultures, with no evidence of polymicrobial infection. Prophylactic dose low‐molecular‐weight heparin (40 mg daily, subcutaneously) was used for the internal jugular vein thrombus after multidisciplinary discussion. He was transitioned to continuous‐infusion ceftriaxone (4 g / 24 h) in the Hospital in the Home program, with a planned six‐week course. The diagnosis of Lemierre's syndrome and base‐of‐skull osteomyelitis caused by *Arcanobacterium haemolyticum* , complicated by ceftriaxone‐associated cholecystitis, was made. Following the development of cholecystitis, ceftriaxone was discontinued and replaced with intravenous benzylpenicillin to complete six weeks of intravenous therapy, followed by six weeks of oral amoxicillin. No formal guidelines exist for the antibiotic duration in skull‐base osteomyelitis; however, the available literature consistently supports a minimum of six weeks of antimicrobial therapy, with many series reporting total durations of 12 weeks or longer [14, 15]. By analogy, the IDSA Clinical Practice Guidelines for Native Vertebral Osteomyelitis, the closest guideline applicable to haematogenous bony infection of the axial skeleton, recommend six weeks of antibiotic therapy as the standard course [16]. On this basis, six weeks of intravenous therapy was selected to ensure adequate bone penetration during the active phase of infection. The subsequent six‐week oral step‐down to amoxicillin was supported by its high oral bioavailability [17].

## 4 Conclusion and Results

At six week review, headaches and cranial nerve palsies had resolved, neck range of motion was normal, and there were no systemic symptoms. Mild left cervical discomfort resolved by eight weeks post discharge. Blood tests were normal. At six month follow up, he remained clinically well with no relapse.

## 5 Discussion

There appears to be a bimodal distribution of hosts in whom *Arcanobacterium* infection occurs. The primary being immunocompetent adolescents particularly, young adult males, with an upper respiratory tract infection and the latter being immunocompromised older adults with skin and soft tissue infections [1, 5]. This is in keeping with our patient's demographics. The latter group of patients have typically been described as having polymicrobial infection [1]. There is a growing body of case reports describing bacteraemia and sepsis in the setting of this pathogen [1, 3]. This almost always seeds from a primary pharyngeal infection [1, 5]. Radiologically, this is in keeping with our case with the MRI demonstrating extensive inflammatory change around the adenoid and palatine tonsils, with associated enhancing soft tissue oedema within the retropharyngeal space. Osteomyelitis is rarely mentioned in the literature, with only three case reports to date, and only one in an immunocompetent host [1, 18, 19, 20]. The immunocompetent host previously described by Stone et al. demonstrated predominately necrotising orbital infection [19]. Brain abscesses have also been described as a complication of *Arcanobacterium* bacteraemia [1]. To date, it seems this is the first reported case of concomitant Lemierre's syndrome and base of skull osteomyelitis. A search of PubMed/MEDLINE using the terms ‘*Arcanobacterium haemolyticum’*, ‘Lemierre's syndrome’, ‘osteomyelitis’, and ‘skull‐base osteomyelitis’ identified no prior reports of these two conditions occurring concomitantly due to this organism.

Most classes of antimicrobials remain efficacious in the treatment of *Arcanobacterium* including cephalosporins, macrolides, aminoglycosides, tetracyclines, and carbapenems [3, 6, 19]. Of note, treatment failure with penicillins has been reported, with the proposed mechanism being *Arcanobacterium's* ability to effectively hide intra‐cellularly [21]. Alrwashdeh et al. reported a mean antimicrobial duration of 36 days in their case report and literature review [1].

There is conjecture in the literature regarding anticoagulation in Lemierre's syndrome. Recommendations differ, from nil anticoagulation to warfarin, and a number of durations suggested [22, 23]. In a recently published meta‐analysis, Gore reported an Odds Ratio (OR) for anticoagulation and mortality of 0.6 and an OR for vessel recanalization and anticoagulation of 1.6, with neither result being statistically significant [24]. A number of case series detail various anticoagulation strategies but none are adequately powered to infer recommendations [24]. Considering the individual's risk and benefit of anticoagulation on a case‐by‐case basis is likely the most judicious approach at this point in time. The risk of bleeding and in particular, intracranial hemorrhage, should be weighed against thrombotic complications [22, 23, 24]. In the absence of risk factors for bleeding or anticoagulation complications, cases with a sequelae of vessel thrombosis despite aggressive antibiotic treatment should be considered candidates for anticoagulation [24]. There are also scant case reports of vessel ligation and excision, despite there being no literature examining the mortality effect of these interventions [24]. It would appear that these interventions are most likely to be considered in patients refractory to, and declining on, medical therapy [24, 25].

Cholelithiasis and cholecystitis are rare complications of ceftriaxone use, especially in adult patients [26, 27]. Cholecystectomy is the definitive therapy for severe cases, though it is thought largely avoidable with interruption of therapy [28]. Typically, ceftriaxone pseudolithiasis affects pediatric populations, though there are case reports of adult patients [9]. There is an isolated case report of the condition being complicated by additional pancreatitis [28]. Proposed risk factors for ceftriaxone‐induced cholecystitis include prolonged therapy, high daily dose, bed rest, impaired gallbladder emptying, and fasting [27, 28, 29]. Renal dysfunction is a reported independent risk factor in adults [29]. A number of potential mechanisms are proposed, including at least in part the high biliary excretion of ceftriaxone at approximately 45% [8, 30]. A proposed contributory gene polymorphism has also been reported in the literature [31]. In our case, high daily dose and prolonged therapy were thought to be contributory factors.

This report details a rare case of both osteomyelitis and Lemierre's syndrome in an immunocompetent host with *Arcanobacterium haemolyticum* bacteraemia. This was further complicated by ceftriaxone induced cholelithiasis and cholecystitis necessitating cholecystectomy.

## Author Contributions

**Angus Lane:** conceptualization, investigation, writing – original draft, methodology, writing – review and editing, project administration. **Alexandra Stewart:** conceptualization, investigation, writing – original draft, writing – review and editing, supervision.

## Funding

The authors have nothing to report.

## Ethics Statement

The patient provided written informed consent and therefore no further ethics review was required.

## Consent

The patient provided written informed consent for the publication of this case report.

## Conflicts of Interest

The authors declare no conflicts of interest.

## Data Availability

Research data are not shared.
